# Differential Effects of Tacrolimus versus Sirolimus on the Proliferation, Activation and Differentiation of Human B Cells

**DOI:** 10.1371/journal.pone.0129658

**Published:** 2015-06-18

**Authors:** Opas Traitanon, James M Mathew, Giovanna La Monica, Luting Xu, Valeria Mas, Lorenzo Gallon

**Affiliations:** 1 Department of Medicine-Nephrology, Northwestern University, Chicago, IL, United States of America; 2 Comprehensive Transplant Center, Northwestern University, Chicago, IL, United States of America; 3 Department of Medicine-Nephrology, Thammasart University Hospital, Pathumthani, Thailand; 4 Department of Surgery, Northwestern University, Chicago, IL, United States of America; 5 Department of Microbiology-Immunology, Northwestern University, Chicago, IL, United States of America; 6 University of Virginia, Department of Surgery, Charlottesville, VA, United States of America; The Jackson Laboratory for Genomic Medicine, UNITED STATES

## Abstract

The direct effect of immunosuppressive drugs calcineurin inhibitor (Tacrolimus, TAC) and mTOR inhibitor (Sirolimus, SRL) on B cell activation, differentiation and proliferation is not well documented. Purified human B cells from healthy volunteers were stimulated through the B Cell Receptor with Anti-IgM + anti-CD40 + IL21 in the absence / presence of TAC or SRL. A variety of parameters of B cell activity including activation, differentiation, cytokine productions and proliferation were monitored by flow cytometry. SRL at clinically relevant concentrations (6 ng/ml) profoundly inhibited CD19^+^ B cell proliferation compared to controls whereas TAC at similar concentrations had a minimal effect. CD27^+^ memory B cells were affected more by SRL than naïve CD27^-^ B cells. SRL effectively blocked B cell differentiation into plasma cells (CD19^+^CD138^+^ and Blimp1^+^/Pax5^low^ cells) even at low dose (2 ng/ml), and totally eliminated them at 6 ng/ml. SRL decreased absolute B cell counts, but the residual responding cells acquired an activated phenotype (CD25^+^/CD69^+^) and increased the expression of HLA-DR. SRL-treated stimulated B cells on a per cell basis were able to enhance the proliferation of allogeneic CD4^+^CD25^−^ T cells and induce a shift toward the Th1 phenotype. Thus, SRL and TAC have different effects on B lymphocytes. These data may provide insights into the clinical use of these two agents in recipients of solid organ transplants.

## Introduction

The evolution of immunosuppressive therapies in transplantation in the past two decades has led to lower rejection rates and improved short-term recipient and allograft outcomes. However, long-term improvement in allograft-survival is still to be achieved [1]. One of the main reasons behind this is the failure of Calcineurin inhibitors (CNI), the cornerstone in the maintenance phase of immunosuppression, to achieve adequate control of the acute and chronic B-cell mediated rejections [2]. To address this problem, a number of immunosuppressive agents have and are being developed to target B cells, plasma cells or antibody production. A number of these agents were used initially for the treatment of B cell or plasma cell malignancies, but later were adopted for use in controlling B cell mediated injury in transplantation. Rituximab, a chimeric anti-CD20 monoclonal antibody, has been shown in a number of studies to have some benefits in the treatment of acute antibody mediated rejection [3–7] but the effect was not confirmed in a recent multicenter, randomized placebo-controlled trial [8]. Recent studies also suggested benefit from Bortezomib, (a proteasome inhibitor that targets plasma cells) [9–12] and Eculizumab (a monoclonal antibody against complement C5) [13, 14] but overall data to support the routine use of these agents in acute and chronic antibody mediated rejection is still weak. While acute antibody mediated rejection is currently manageable with variable success, chronic antibody mediated rejection is even more difficult to treat because of irreversible damage that has already occurred in the allografts [15].

Although a lot of current studies are focused on investigating new immunosuppressive agents that target B cells, current understandings of the effect of conventional immunosuppressive drugs on B cells are still limited because they were primarily designed to target T cells and prevent acute cellular rejection. Cyclosporine and TAC were shown to have varying effects on B lymphocyte proliferation depending on the dosage and type of stimulation [16–18] but the effect on T cell-independent antibody production was inconclusive [16, 19–21]. In contrast, SRL has been shown to inhibit B cell proliferation, decrease both T cell-dependent and T cell-independent antibody production and also increase B cell apoptosis [16, 19–21]. However, no data are available on the effects of these conventional immunosuppressive drugs on B cell subpopulations and differentiation.

In this study, we questioned whether TAC and SRL at clinically relevant concentrations affect CD19^+^ B cell activation, proliferation and differentiation. It was observed that SRL inhibited proliferation and differentiation into plasma cells, but increased the percentage of cells expressing CD25, CD69 and HLR-DR in the residual responders, when compared to control or TAC. Furthermore, SRL-treated stimulated B cells on a per cell basis were able to mediate amplified alloreactivity in CD4^+^CD25^−^ T cells towards the Th1 phenotype.

## Materials and Methods

### Subjects

Written informed consent was obtained from each subject, and research protocols were approved by the Institutional Review Board of Northwestern University (IRB # STU00002452) in accordance with regulations mandated by the Department of Health and Human Services.

### Isolation of B cells

Blood was obtained from healthy volunteers after informed consent. Peripheral blood mononuclear cells (PBMC) were isolated by Ficoll Hypaque density gradient centrifugation. Total B cells were isolated from PBMC by positive selection using human CD19 MicroBeads kit (MACS, Miltenyi Biotec, Auburn, CA). Naïve and memory B cells were isolated from total B cells by human CD27 MicroBeads kit (Miltenyi Biotec).

### Culture Conditions

B-cells were cultured in 96-well plates (1-2x10^5^/well / 200ul medium) at 37°C in a 5% CO_2_ humidified incubator in medium consisting of Iscove's Modified Dulbecco's Medium (IMDM) (Invitrogen, Carlsbad, CA) supplemented with 10% human serum AB (Gemini Bio-products, West Sacramento, CA), 0.5 ml Human Insulin (Sigma-Aldrich, St. Louis, MO), 200mM L-Glutamine (Sigma-Aldrich) and Gentamycin (Invitrogen). They were stimulated through the B cell receptor with 5ug/ml anti-IgM (Jackson Immuno Research, West Grove, PA) in presence of 100ng/ml anti-CD40 (R&D systems, Minneapolis, MN) and 100 ng/ml IL-21 (PEPROTECH, Rocky Hill, NJ) [22]. Tacrolimus or Sirolimus (Axxora, San Diego, CA) was added to the cell cultures at different concentrations (2 to 6 ng/ml); the controls consisted of cultures without the drugs. The choices of these concentrations of TAC and SRL were based on our extensive experience in using them at a wide range of concentrations in similar *in vitro* culture system, albeit on T cell responses as previously reported [23, 24] [and Levitsky et.al. submitted]. The culture duration was for 6 days.

### Flow cytometry and CFSE labeling

After 6 days culture, cultured cells were analyzed for expression of surface markers by multi-color flow cytometry. Cell aliquots were treated with anti-human Fc mAb for 20 minutes and stained for 30 minutes with selected combinations of fluorochrome-conjugated antibodies.

For the analysis of B cell subpopulations and activation markers, the monoclonal antibodies used were CD19-PE-CF594 (BD Biosciences, San Jose, CA), CD62L-PE-Cy5 (BD Biosciences), IgD-FITC (BD Biosciences), CD27-PE-Cy7 (BD Biosciences), CD24-PE (BD Biosciences), CD38-PerCP-Cy5.5 (Beckman Coulter, Brea CA), CD138-PE-Cy7 (eBioscience, San Diego, CA), CD23-PE (BD Biosciences), CD21-PE-Cy5 (BD Biosciences), IgG-PC7 (BD Biosciences), CD86-Alexa 700 (BD Biosciences) and CD95-Pacific Blue (BioLegend, San Diego, CA).

For plasma cell staining, cultured cells were first incubated with monoclonal antibodies to surface markers (CD38, CD138 and CD19), fixed and permeabilized with Cytofix/Cytoperm Fixation/Permeabilization Solution Kit (BD Biosciences) and then incubated with Blimp-1-PE (R&D systems) and Pax5-APC (eBioscience) for 30 minutes.

For the proliferation assay, 1–10×10^6^ cells of interest were labeled with 1μM carboxyfluorescein succinimidyl ester (CFSE) (Invitrogen) before culture initiation. After 6 days of culture, cells were harvested, incubated with antibodies, and dilution of CFSE was assessed by flow cytometry.

### *In vitro* T-cell proliferation assay

CD4^+^CD25^−^ T cells were isolated from PBMC by 2 steps separation of depletion using human CD25 MicroBeads and positive selection with human CD4 MicroBeads (Miltenyi Biotec). After labeling with CFSE these responder cells (5x10^4^/well) were stimulated with irradiated B cells (1x10^5^/well) that had been pre-stimulated with α-IgM and α-CD40 mAb plus IL-21 in the absence or presence of SRL or TAC for 6 days. After an additional 6 days in culture, these MLRs were harvested and the proliferation was determined by assessing CFSE dilution.

For the analysis of T cell subpopulations and activation markers, harvested MLR cultures were subjected to flow cytometry using CD25-PE (BD Biosciences), CD62L-PE-Cy5 (BD Biosciences), CD4-ECD (Beckman Coulter), CD45RO-PC7 (BioLegend), CD69-PE-Cy5 (BD Biosciences), CD19-PE (BD Biosciences), CD3-ECD (Beckman Coulter) and CD95-Pacific Blue (BioLegend).

For the intracellular cytokine or transcription factor estimation, cultured cells were stimulated with 20ng/ml PMA and 500 ng/ml ionomycin for 6 hours in presence of GolgiStop (BD Biosciences) during the last 4 hours of incubation to prevent cytokine secretion. Then the cells were first stained for surface markers, fixed and permeabilized with a BD Cytofix/Cytoperm Fixation/Permeabilization Solution Kit (BD Biosciences) and incubated with selected combination of fluorochrome conjugated antibodies: IFN-γ-APC-e780 (eBioscience), IL-10-FITC (BioLegend), IL-17-PE (BioLegend), GATA3-FITC (BioLegend) and Foxp3-PE (eBioscience), IL-4-APC Biolegend (BioLegend), IL-2-PERCP-Cy5.5 (BioLegend), T-bet V450 (BD Biosciences) and RORγt-APC (eBioscience).

The stained samples were analyzed on a Cytomics FC500 Flow cytometer (Beckman Coulter) or a BD LSR Fortessa (BD Biosciences) and the data were analyzed by CXP software version 2.2 (Beckman Coulter) or FlowJo software version 9.4.5 (Tree Star, Inc. Ashland, OR).

### Cytokine assays

Supernatants were collected on day 6 of cultures and stored at -20°C. The concentration of the cytokines were measured by MILLIPLEX MAP Human Cytokine/Chemokine Magnetic Bead kit (EMD Millipore, Billerica, MA).

### Statistical analysis

All analyses were performed by SAS software version 9.2 (SAS Inc., Cary, NC). Values are shown as mean ± SD. Statistical calculations were made using the standard t test for independent samples (two-tailed) or Wilcoxon rank test for continuous data that were not normally distributed. Two-sided p values < 0.05 were considered to indicate statistical significance.

## Results

### SRL, but not TAC, effectively inhibited B cell proliferation

To assess the effect of SRL and TAC on the proliferative capacity of B cells, CD19^+^ cells were isolated from PBMC by positive selection using human CD19 MicroBeads kit. The purity of CD19+ cells was more than 95%. Freshly isolated B cells were labeled with CFSE and then stimulated with anti-IgM, anti-CD40 mAb and IL-21 in the presence or absence of SRL and TAC for 6 days. The cells were then harvested and CFSE dilution was assessed by Flow cytometry.

**Fig 1:**
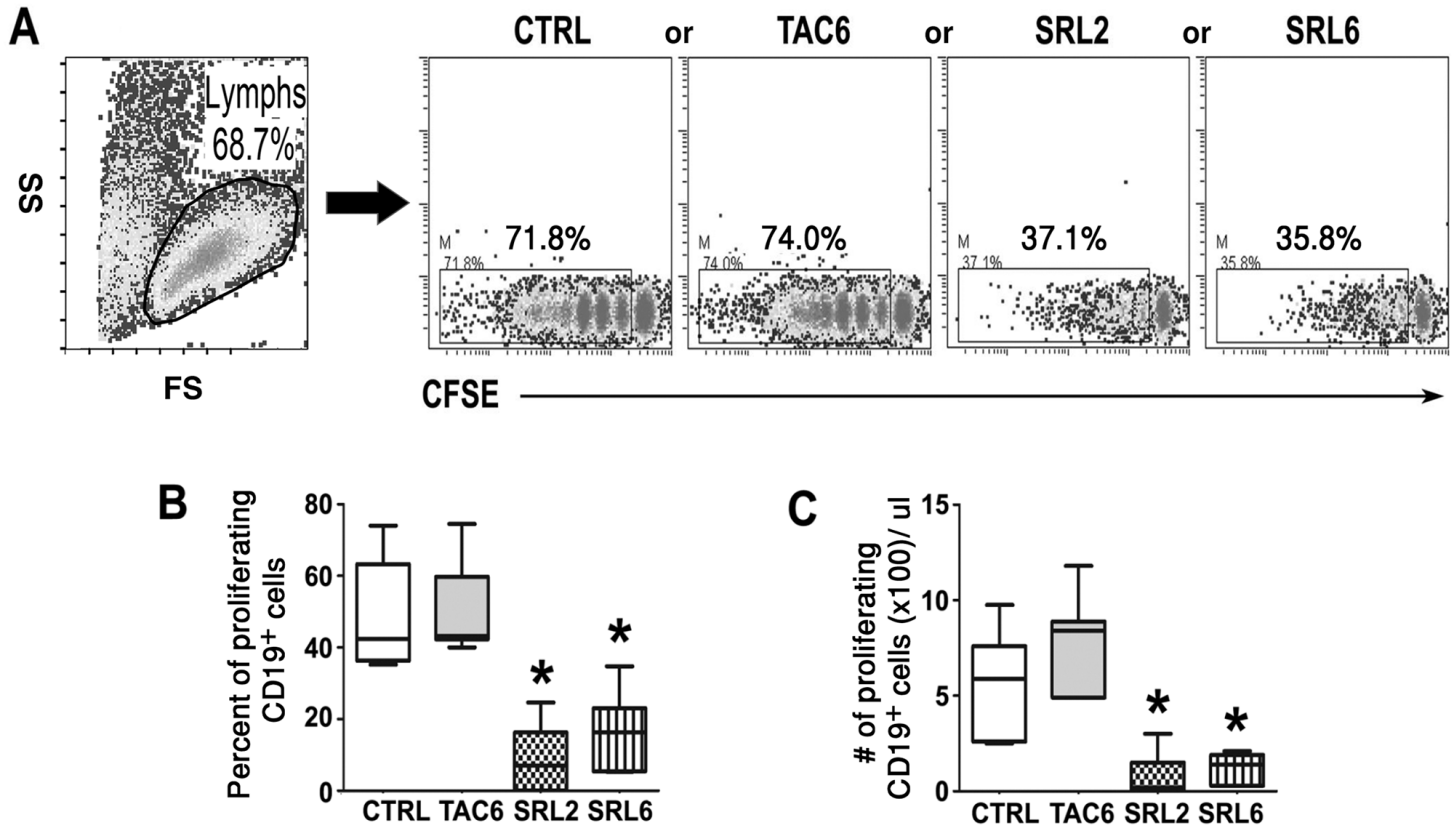
SRL effectively inhibited B-cell proliferation. Purified CD19^+^ B cells were labeled with CFSE, stimulated with anti-IgM, anti-CD40 mAb and IL-21 (BCR method) in the absence (control; CTRL) or presence of TAC (6ng/ml) or SRL (2ng/ml or 6ng/ml) and flow cytometric analyses were performed after 6 days in culture. **(A)** A representative experiment: cells were gated on viable lymphocytes and analyzed for CFSE diluting proliferating cells. This scheme of analysis was used in all subsequent experiment, unless indicated otherwise. **(B)** The percentage of proliferating CD19^+^ B cells as obtained in A from 7 different experiments. **(C)** Absolute number of proliferating CD19^+^ B cells was calculated in each experiment by multiplying the recovered cell counts with the percentage of proliferating cells as in A (n = 7). Statistically significant (*p < 0.05) inhibition of B cell proliferation was observed with SRL at both subtherapeutic (2ng/ml) and therapeutic (6ng/ml) doses.

After 6 days of stimulation, more than 70% B cells proliferated in the control cultures without the drugs (Fig 1A and 1B). TAC at clinically relevant dose of 6 ng/ml did not have any inhibitory effect on B cell proliferation. In contrast, in the SRL treated cultures, the percentage of proliferating cells was significantly reduced even at subtherapeutic 2 ng/ml. Concomitantly, the absolute numbers of proliferating CD19^+^ B cells were also significantly reduced in the SRL treated cultures when compared to those in the controls or the TAC treated cultures (Fig 1C).

### B cell stimulation in the presence of SRL resulted in a population shift toward an activated phenotype

**Fig 2:**
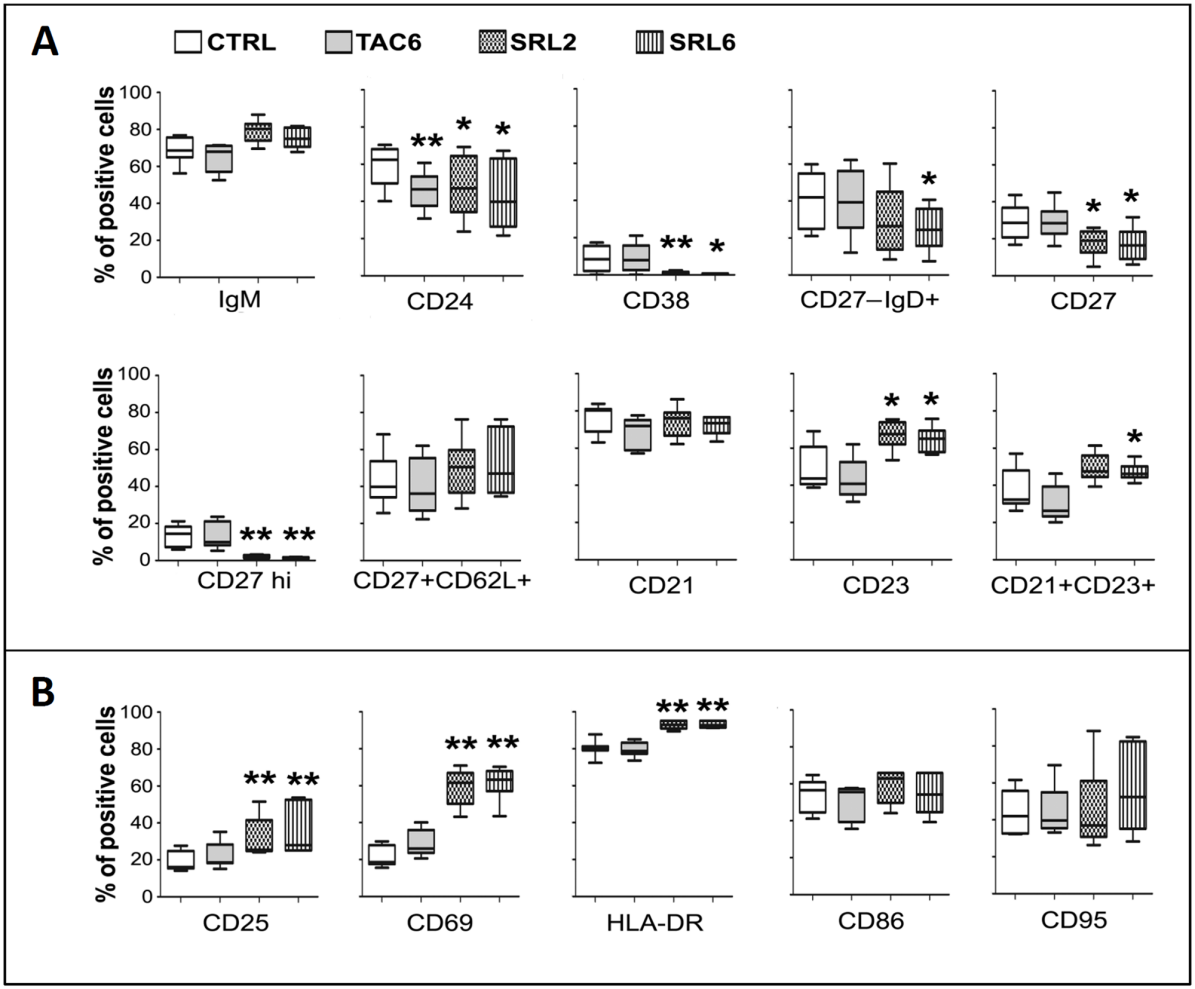
B-cell stimulation in the presence of SRL resulted in a population shift toward an activated phenotype. Purified CD19^+^ B cells were stimulated with anti-IgM, anti-CD40 mAb and IL-21 and multi-color flow cytometric analyses were performed on day 6 as described in Fig 1. The figures **A**, **B** and **C** show the expression of various surface markers on stimulated B cells (Percentage of positive cells/total proliferating CD19^+^ cells) p < 0.05, **p < 0.01. CTRL, control; TAC6, 6 ng/ml TAC; SRL2, 2 ng/ml SRL; SRL6, 6 ng/ml SRL.

Subsequently, we studied the phenotypic make-up of the proliferating B cells in the control, TAC and SRL treated cultures by flow cytometry (Fig 2). Both the control and TAC-treated cultures had similar subset profile; the only significant difference was a decreased percentage of immature CD24^+^ B cells with TAC, suggesting that TAC did not favor or inhibited their expansion. In contrast, residual B cells that proliferated in the presence of SRL had an entirely different subset profile. There were significantly lower percentages of immature CD24^+^ and CD38^+^ B cells [25, 26], lesser naïve CD27^-^IgD^+^ cells (especially at 6ng/ml SRL; p = 0.022), and reduced CD27^+^ total memory B cells or even memory B cells that differentiated into plasma cells (CD27^hi^ B cells) (p<0.05, p<0.01 compared to TAC or control group, respectively). However, no significant difference was observed among the three different cultures in the percentages of memory B cells expressing the trafficking receptor CD62L (CD27^+^CD62L^+^) or the complement receptor 2 (CD21). The B cells that proliferated in presence of SRL, however, had significantly higher percentage of cells that expressed CD23, a low affinity IgE receptor present on mature/activated B cells [27–29] and a subset of cells with the follicular B cell phenotype (CD21^+^CD23^+^ B cells) [30].

Although the B cells cultured in presence of SRL had significantly lower proliferation (Fig 1), among the residual cells that did proliferate higher percentages expressed the activation markers CD25, CD69 and HLA-DR (Fig 2C) when compared to those in both the control and TAC groups (p<0.01). However, the percentages of cells expressing the activation markers CD86 and CD95 were at comparable levels.

### SRL but not TAC inhibited the proliferation of CD19^+^CD27^−^ naïve and CD19^+^CD27^+^ memory B cells

**Fig 3:**
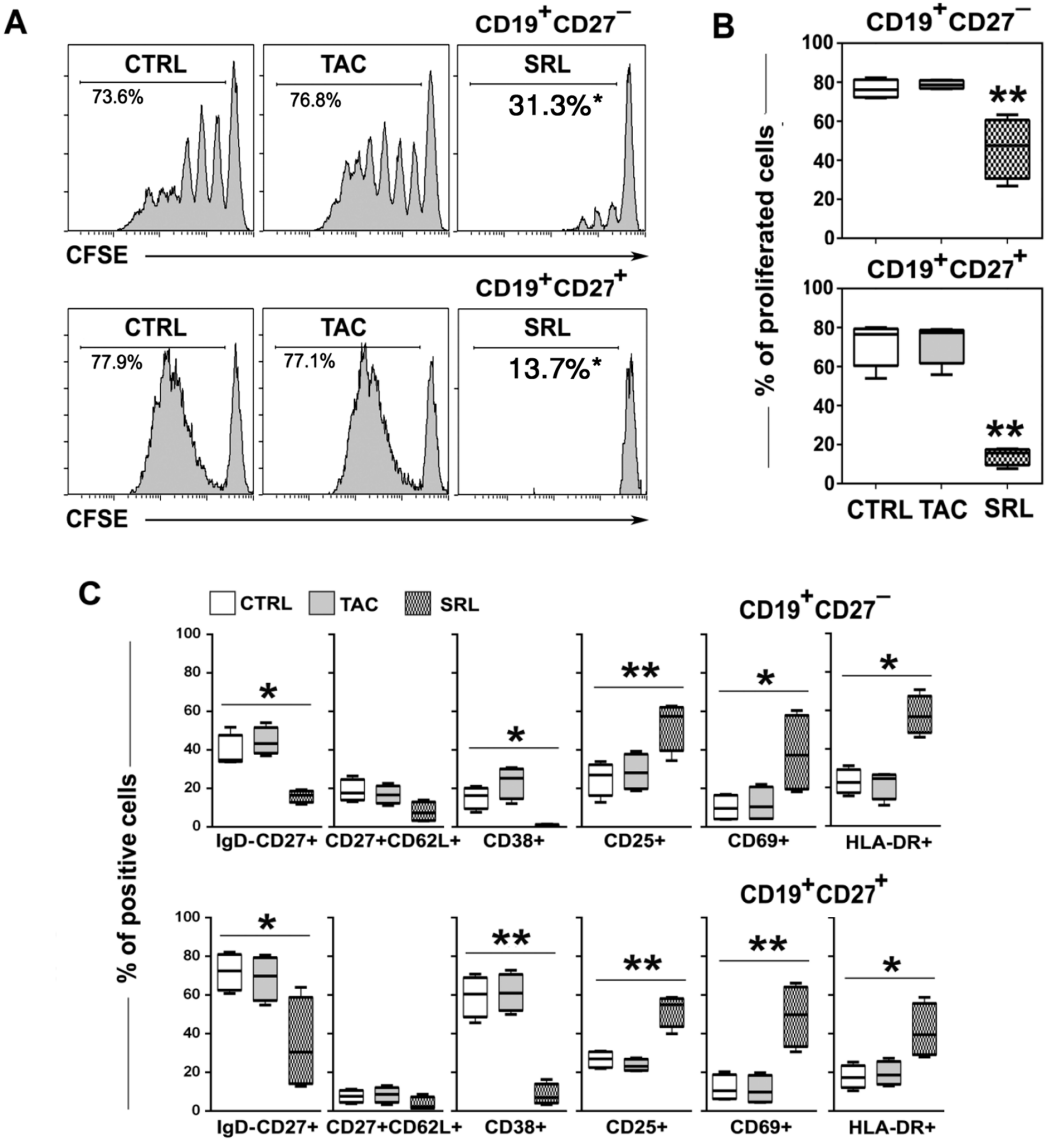
SRL but not TAC inhibited the proliferation of CD19^+^CD27^−^ naïve and CD19^+^CD27^+^ memory B cells. B cells were purified by depleting non-B cells resulting in >95% CD19^+^ cells which subsequently sorted into CD27^−^ (naïve) and CD27^+^ (memory) B-cell fractions. These subsets were labeled with CFSE, stimulated with anti-IgM, anti-CD40 mAb and IL-21 in the absence (CTRL) or presence of TAC or SRL at 6ng/ml and were analyzed by multicolor flow cytometry after 6 days in culture. **(A)** A representative experiment: histogram plots show dilution of CFSE in the proliferating CD19^+^CD27^−^ (upper panel) or CD19^+^CD27^+^ (lower panel) cells. **(B)** Data are from four different independent experiments are shown as mean ± SD percent proliferating naïve CD19^+^CD27^−^ and memory CD19^+^CD27^+^ B cells. **(C)** B cell subsets showing indicated surface markers were analyzed and plotted as mean ± SD (n = 4) percent of proliferating cells in the cultures of naïve CD19^+^CD27^−^ (upper panel) and memory CD19^+^CD27^+^ B cells (lower panel). The residual cells that proliferated in presence of SRL demonstrated an activated phenotype. *p < 0.05, **p < 0.01.

To further analyze the differential effects of TAC and SRL on naïve and memory B cells, CD19^+^CD27^-^ (naïve) and CD19^+^CD27^+^ (memory) B cells were sorted from PBMC and tested in culture. The purity of the sorted cells was more than 95%. After 6 days in culture under the 3 conditions, the cells were analyzed for CFSE dilution and phenotypic makeup by flow cytometry. It was observed that TAC had only minimal effect on the proliferation of both memory CD19^+^CD27^+^ and naïve CD19^+^CD27^-^ B cells (Fig 3A and 3B). Interestingly, however, SRL was found to be more effective in inhibiting the proliferation of memory CD19^+^CD27^+^ B cells than that of the naïve CD19^+^CD27^-^ B cells. Additionally, SRL prevented the differentiation of CD27^-^ B cells into CD27^+^ subsets that were negative for surface IgD or into CD38^+^ cells when compared to the control or TAC (Fig 3C, top row). Similarly, SRL significantly inhibited (p<0.05) the expansion of these subsets in the starting cultures of CD27^+^ B cells also ((Fig 3C, bottom row). However, compared to control and TAC, SRL significantly amplified the percentage of cells that expressed the activation markers CD25 and CD69 as well as upregulated the expression of HLA-DR in both CD27 positive and negative responder B cells after 6 days in culture. These results with the subsets confirmed our observations with total CD19^+^ cells shown above in Fig 2 that stimulation of B cells in the presence of SRL would result in a population shift toward an activated phenotype.

### SRL, but not TAC, effectively inhibited the differentiation of B cells into plasma cells

**Fig 4:**
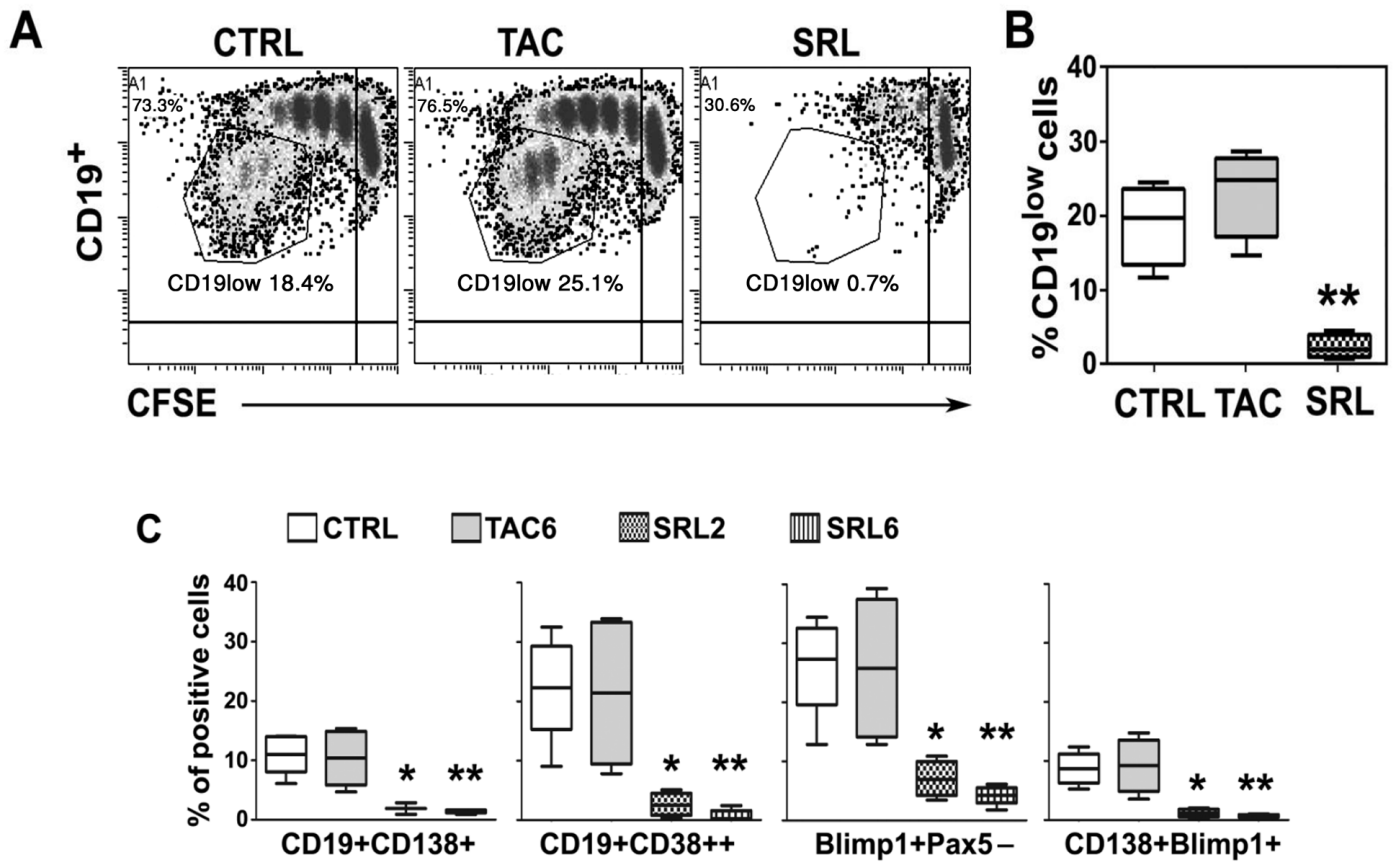
SRL, but not TAC effectively inhibited the differentiation of B cells into plasma cells. Purified CD19^+^ B cells were cultured as in Figs 1 and 2. **(A)** A representative experiment showing flow cytometric profile indicative of putative plasma cells (CD19^low^) in proliferated B cells (gated on viable lymphocytes; see Fig 1A). **(B)** The mean ± SD percentage of such CD19^low^ B cells from 4 different independent experiments. **(C)** Mean ± SD (n = 4) percentage of CD19^low^CD38^++^ plasmablasts, CD19^low^CD138^+^ plasma cells, Blimp1^+^PAX5^-^ cells and CD138^+^Blimp1^+^ cells in the proliferating CD19^low^ cells. *p < 0.05, **p < 0.01. CTRL, control; TAC, 6 ng/ml TAC; SRL, 6 ng/ml SRL.

CFSE labeled B cells were stimulated as above for 6 days and were monitored for differentiation into plasma cells by flow cytometry. When compared to the controls and TAC treated cultures, the SRL treated cultures had significantly lower proliferating cells as well as cells that down-regulated the CD19 marker (Fig 4A and 4B). Additionally, the SRL-treated cultures had significantly lower percentages of CD19^low^CD38^++^ plasmablasts and CD19^low^CD138^+^ plasma cells (Fig 4C). Similarly, when the intracellular expressions of protein B lymphocyte-induced maturation protein 1 (BLIMP1) and paired box protein 5 (PAX5) were investigated, the proportion of CD19^low^ cells with antibody-secreting phenotype, Blimp1^+^PAX5^-^ and CD138^+^Blimp1^+^ were markedly reduced in the SRL-treated cultures. These results confirmed that SRL would block the differentiation of B cells into plasma cells.

### SRL was more effective in inhibiting cytokine production in proliferating B cells

**Fig 5:**
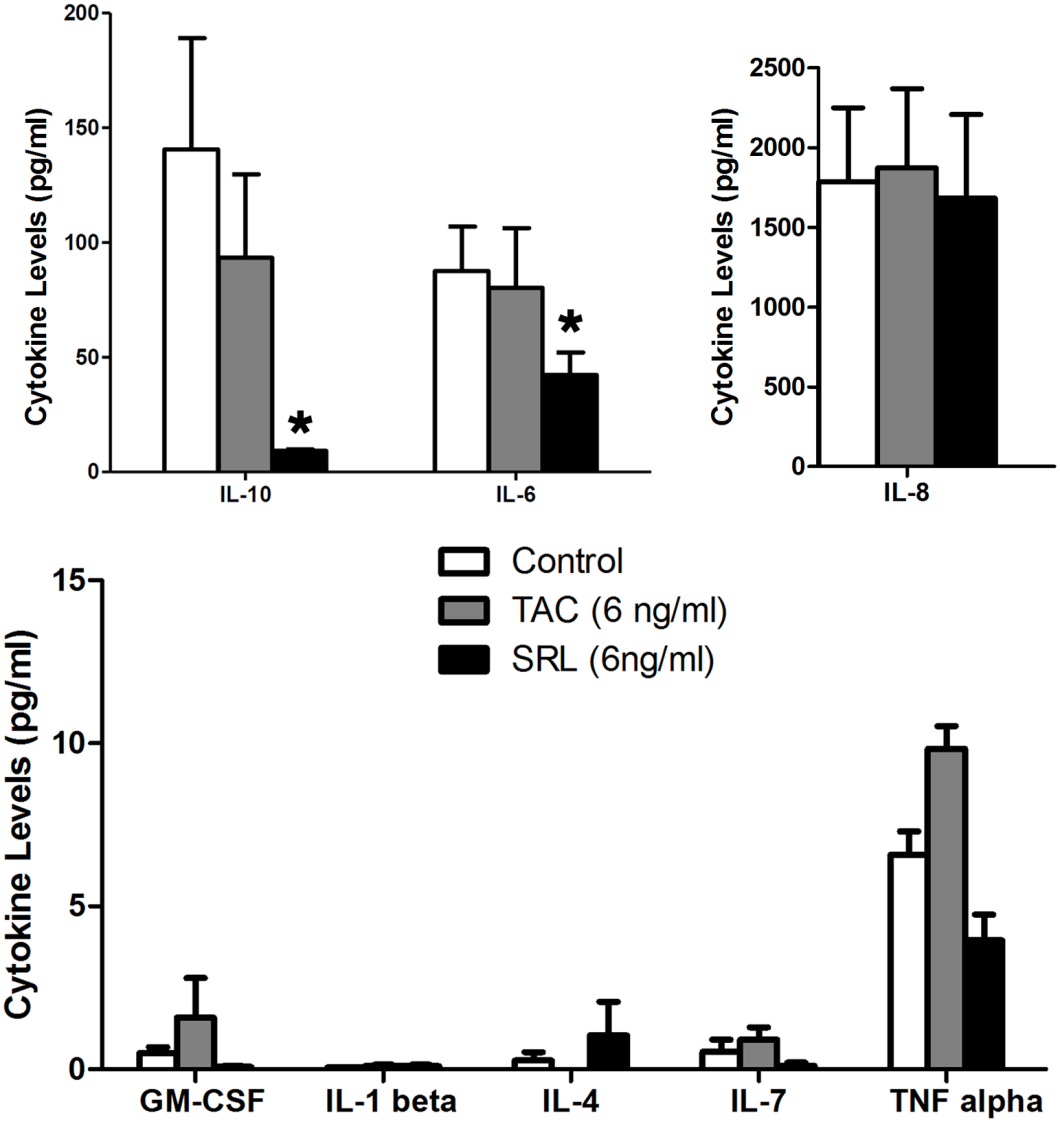
SRL was more effective in inhibiting IL-6 and IL-10 production in B cells. Cytokine levels were measured in the culture supernatants of stimulated B cells in the absence or presence of 6ng/ml SRL or TAC on day 6. SRL markedly inhibited production of IL-10 and IL-6 by B cells compared to control and TAC. No significant difference was found for IL-1beta, IL-4, IL-7, IL-8, TNF-alpha and GM-CSF. *p < 0.05, **p < 0.01.

To examine if the B-cell population shift induced by SRL was associated with changes in their cytokine production, supernatants from stimulated B cell cultures were collected on day 6 and the levels of IL-1beta, IL-4, IL-6, IL-7, IL-8, IL-10, TNF-alpha and GM-CSF were measured. It was observed that SRL markedly inhibited production of IL-10 and IL-6 by B cells compared to control and TAC (Fig 5). No significant difference was found for IL-1beta, IL-4, IL-7, IL-8, TNF-alpha and GM-CSF.

### SRL-treated B cells enhanced the proliferation and differentiation of CD4^+^ T cells

**Fig 6:**
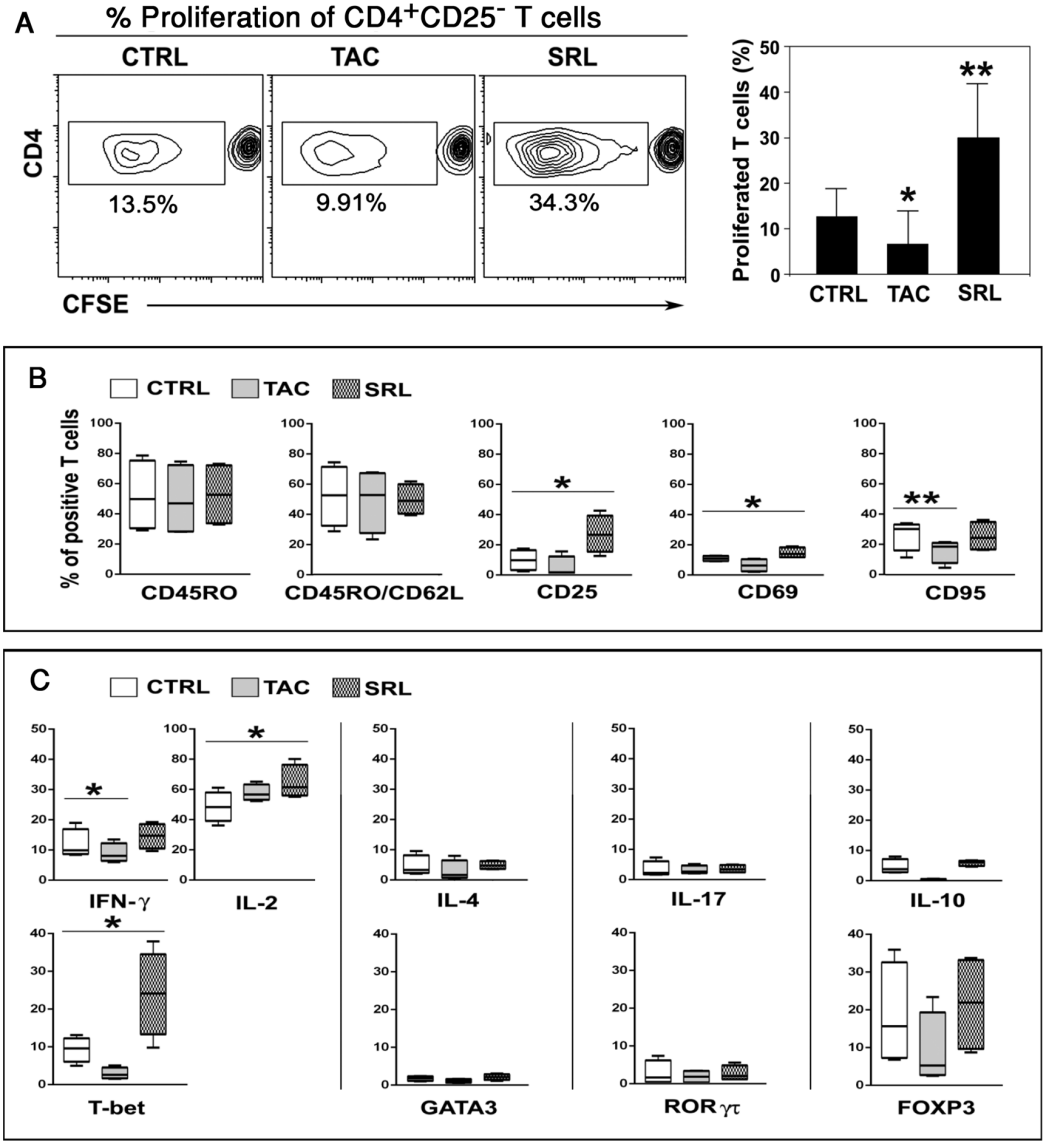
SRL-treated B cells enhances the proliferation and differentiation of CD4^+^ T cells. Purified CD19^+^ B cells were pre-stimulated for 6 days with anti-IgM, anti-CD40 mAb and IL-21 in the absence (CTRL) or presence of 6ng/ml TAC or SRL. These cultured B cells were used as stimulators in 6-day MLRs of allogeneic CFSE-labelled CD4^+^CD25^−^ T cell responders. (**A**) Level of proliferation differentially induced by pre-cultured B cells as detected by CFSE dilution in the allogeneic CD4 responder cells (representative experiment on the left, and compiled data from 8 independent experiments on the right). (**B**) Percentage of responding T cells positive for memory marker (CD45RO) and activation markers (CD62L, CD25, CD69, CD95) after co-culture with pre-stimulated B cells (n = 8); (**C**) Mean ± SD (n = 4) percentage of responding proliferating T cells expressing intracellular cytokines (top row) and transcription factors (bottom row). Taken together the data indicated that B cells that proliferated in presence of SRL on a per cell basis were capable of inducing alloreactive T cell proliferation towards a Th1 phenotype. *p < 0.05. ** p < 0.01.

Since the SRL-treated B cells had higher proportion of HLA-DR expressing cells, the antigen presenting function of these cells was tested. B cells were stimulated in the absence or presence of TAC or SRL for 6 days, and were used as stimulators in primary MLRs with CFSE-labeled allogeneic CD4^+^CD25^−^ responder T cells. B cells grown in presence of SRL were able to induced significantly more T cell proliferation than B cells from control or TAC-treated cultures (p<0.01) (Fig 6A). Phenotypic analysis of the proliferating T cells further revealed that SRL-treated B cells also induced higher proportion of cells expressing activation markers CD25 and CD69 but not CD95 (Fig 6B). No difference in the percentage of memory (CD45RO^+^) T cells was observed among the three groups.

Intracellular transcription factor and cytokine staining showed that the proliferating T cells induced by SRL-treated B cells also had significantly higher proportion of cells expressing T-bet, IL-2 and IFN-γ (Fig 6C) compared to the controls or TAC treated B cells. No differences were observed on the percentages of Th2 (IL4 and GATA3), Th17 (IL-17 and RORγt) and Treg (IL-10 and FOXP3) positive cells among the three (Fig 6C). Taken together, these data suggested that the B cells that proliferated and differentiated in presence of SRL on a per cell basis was able to induce higher alloreactivity with a distinct shift toward the Th1 phenotype.

## Discussion

Studies on immunosuppressive drugs in transplantation over the past two decades have mainly been focused on depleting T cells or inhibiting T cell function. However, humoral immunity mediated by B cells, plasma cells and antibodies secreted by them has been increasingly recognized as a player in both acute and chronic graft injury [31]. Aside from being the producers of antibodies, B cells also function as potent antigen presenting cells. Therefore, the need to understand the effects of currently used immunosuppressive drugs on immune responses mediated by B cell is becoming increasingly recognized. Hence, we studied the differential effects of TAC versus SRL on B cells upon stimulation through the B cell receptor and found that TAC had almost no effect on the activation and proliferation of B cells whereas SRL even at subtherapeutic doses inhibited their proliferation (Fig 1) and differentiation into plasma cells (Fig 4). However, the residual B cells that did respond to stimulations in the presence of SRL resulted in a population shift toward activated phenotypes (Figs 2 and 3) which in turn on per cell basis were able to induce a robust allogeneic T cells activation and proliferation towards a Th1 phenotype (Fig 6).

A number of in vitro studies have been performed to investigate the effects of immunosuppressive drugs, especially TAC on human B cells. TAC has been shown to inhibit the proliferative response of resting B cells induced by Staphylococcus aureus Cowan strain I (SAC) and phorbol myristate acetate (PMA) in a dose-dependent manner [18]. TAC and Cyclosporine have been shown to inhibit initial T cell-dependent B cell activation by pokeweed mitogen but failed to inhibit T cell-independent activation by SAC (20), somewhat similar to the observations made in this report. However, once the B cells are activated, they became resistant to inhibition by TAC [20]. TAC is also found to marginally inhibit the B cell proliferation depending on the degree of B cell stimulation [16].

In contrast to the observations with TAC, SRL has consistently been found to have inhibitory effects on B cell activation and proliferation independent of the stimuli tested [16, 32, 33]. Our studies have extended these observations by demonstrating that the residual B cells that did respond to stimulations in the presence of SRL result in a population shift toward activated phenotypes and that they in turn on per cell basis are able to induce a robust allogeneic T cells activation and proliferation towards a Th1 phenotype.

While Rituximab has been recently reported to have the inhibitory effect only on CD19^+^CD27^-^ naïve B cells but not on the proliferation of CD19^+^CD27^+^ memory B cells [34], SRL in our study was able to profoundly inhibit the proliferation of CD19^+^CD27^+^ memory B cells but had relatively less effect on the CD19^+^CD27^-^ naïve B cells (Fig 3A and 3B). Although we know that mTOR regulates most of the events that involve in B cell responses including migration, growth/proliferation and differentiation, little is known regarding how mTOR coordinates these events in B cell responses [35]. Whether the memory B cell activation depends more on mTOR pathway than the naïve B cells remains to be investigated.

Scarce data exist regarding the relative effects of various immunosuppressive drugs including TAC or SRL on *in vivo* antibody production particularly in clinical transplantation. Perusal of available literature does not provide a clear understanding of the differential roles of each of these IS *in vivo* as they are used in combination with other IS drugs. A couple of studies that tangently address this issue has indicated an increased risk for the development of DSA upon conversion early after transplantation from CNI to everolimus, a derivative of SRL [36, 37]. However, there is a consensus that most of the currently used IS combinations do not seem to impact humoral rejection. In fact, current approaches to control DSA are based on mechanical removal of alloantibodies via plasmapheresis and through the use of IVIg with or without rituximab that specifically target CD20 on B cells.

We have also found that SRL is very effective in inhibiting the differentiation of B cells into plasma cells upon stimulation through the B Cell Receptor (BCR) (Fig 4C). Previously SRL has been shown to block the differentiation of B cells into antibody secreting cells when stimulated with polyclonal activator Staphylococcus aureus + IL2 or CD40L + IL-2, by blocking cell cycle progression past mid-G^1^ phase [33]. SRL inhibits the mTOR pathway by directly binding the mTOR Complex1 (mTORC1) [38]. mTORC1 phosphorylates the ribosomal S6 kinases (S6Ks) and the eukaryotic translation initiation factor 4E-binding protein (4EBPs) along with the suppression of subcellular autophagy, are essential for cell growth in preparation for division [39]. In B cells, Phosphorylation of S6Ks and 4EBPs occurs rapidly following B cell receptor engagement [40] but whether these signals are required for successful growth and proliferation of B cells remains unclear [39]. Mice with mTOR deleted in their B cell lineage (knockout) have been shown to produce fewer splenic germinal centers and have decreased high-affinity antibody responses compared to the wild type mice, indicating an important immunoregulatory role of mTOR in the germinal center [41].

Despite the inhibitory effect of SRL on B cell proliferation, we have observed that the remaining B cells that do respond to stimulation in the presence of SRL result in a population shift of B cells toward the activated phenotypes and expressing more HLA-DR. Since these effects present two variables of cell numbers versus stimulatory capacity, vis-à-vis SRL versus control or TAC an accurate measurement of the net effect cannot be made, and hence is a limitation of our study. However, these activated B cells in SRL group on per cell basis are able to induce more proliferation of allogeneic T cells which in turn resulted in a shift of proliferating T cells toward the Th1 Phenotype. These findings may in part be explained further by the recent reports that show that inhibition of mTOR enhances MHC antigen presentation via subcellular autophagy induction in monocytes/macrophages and dendritic cells [42]. Autophagy has been shown to facilitate the presentation of endogenous proteins on MHC class I and class II molecules and leading to activation of CD4+ T cells [43]. mTOR is an important negative regulator of autophagy so SRL which inhibits mTOR can induce autophagy [44].

Emerging evidences show that despite the antibody production, B cells are able to produce both regulatory and effector cytokines and contribute to the disease pathogenesis in an antibody-dependent fashion [45–47]. In this study, we have found that a number of cytokines are produced by B cells upon stimulation. SRL is able to decrease both proinflammatory cytokine IL-6 and anti-inflammatory cytokine IL-10 in the culture supernatant. IL-10 has been shown to stimulates DNA replication of B cells and induce anti-CD40-activated B cells to secrete antibodies [48, 49] while IL-6 is reported to be involved in the final maturation of B cells into antibody secreting cells [50].

In summary, we have shown that the conventional immunosuppressive drug SRL has potent inhibitory effects on B cell proliferation and differentiation whereas TAC has no such effects. However, the residual B cells that do respond to stimulations in the presence of SRL result in a population shift toward activated phenotypes. These activated B cells on per cell basis are able to induce a robust allogeneic T cells activation and proliferation towards a Th1 phenotype. The data generated from this in vitro study can help to elucidate how SRL and TAC affect B cells subpopulation and differentiation and can guide the clinical use of these agents in recipients of solid organ transplants. It may be speculated that while treatment with TAC controls T cell mediated immune responses and to a lesser extend the B cell mediated responses, the opposite may be true with SRL treatment.
